# Assessing Kwa-Zulu-Natal’s progress towards malaria elimination and its readiness for sub-national verification

**DOI:** 10.1186/s12936-019-2739-5

**Published:** 2019-04-01

**Authors:** Ryleen Balawanth, Inessa Ba, Bheki Qwabe, Laura Gast, Rajendra Maharaj, Jaishree Raman, Rebecca Graffy, Mbavhalelo Shandukani, Devanand Moonasar

**Affiliations:** 1 Clinton Health Access Initiative, Pretoria, Gauteng South Africa; 2 KwaZulu-Natal Department of Health, Jozini, KwaZulu-Natal South Africa; 3 South African Medical Research Council, Durban, KwaZulu-Natal South Africa; 4 National Institute of Communicable Disease, Sandringham, Gauteng South Africa; 5 South Africa National Department of Health, Pretoria, Gauteng South Africa

**Keywords:** Malaria, Elimination, Programmatic review, South Africa

## Abstract

**Background:**

The South African province of KwaZulu-Natal is rapidly approaching elimination status for malaria with a steady decline in local cases. With the possibility of achieving elimination in reach, the KZN malaria control programme conducted a critical evaluation of its practices and protocols to identify potential challenges and priorities to achieving elimination. Three fundamental questions were addressed: (1) How close is KZN to malaria elimination; (2) Are all systems required to pursue subnational verification of elimination in place; and (3) What priority interventions must be implemented to reduce local cases to zero?

**Methods:**

Based on the 2017 World Health Organization Framework for Elimination, twenty-eight requirements were identified, from which forty-nine indicators to grade elimination progress were further stratified. Malaria data were extracted from the surveillance system and other programme data sources to calculate each indicator and semi-quantitatively rate performance into one of four categories to assess the provinces elimination preparedness.

**Results:**

Across the key components a number of gaps were elucidated based on specific indicators. Out of the 49 indicators across these key components, 10 indicators (20%) were rated as fully implemented/well implemented, 11 indicators (22%) were rated as partially done/somewhat implemented/activity needs to be strengthened, and 12 indicators (24%) were rated as not done at all/not implemented/poor performance. Sixteen indicators (33%) could not be calculated due to lack of data or missing data.

**Conclusions:**

The critical self-evaluation of programme performance has allowed the KZN malaria programme to plan to address key issues moving forward. Based on the findings from the checklist review process, planning exercises were conducted to improve lower-rating indicators, and a monitoring and evaluation framework was created to assess progress on a monthly basis. This is scheduled to be reviewed annually to ensure continued progress toward meeting the elimination goal. In addition, multiple dissemination meetings were held with both provincial senior management and operational staff to ensure ownership of the checklist and its action plan at all levels.

**Electronic supplementary material:**

The online version of this article (10.1186/s12936-019-2739-5) contains supplementary material, which is available to authorized users.

## Background

**Fig. 1:**
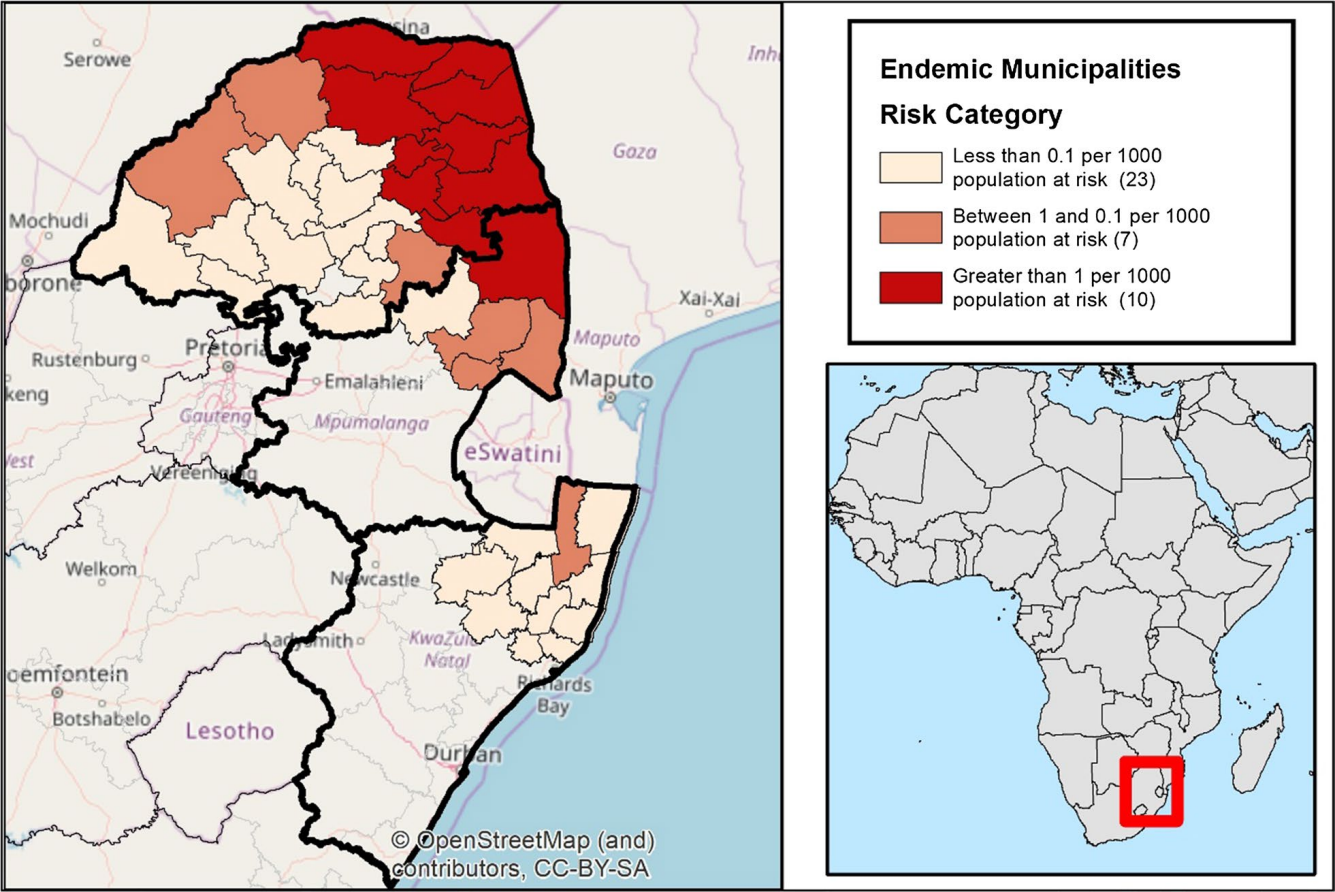
Remaining 40 malaria-endemic municipalities in the 3 remaining endemic Provinces of South Africa, by risk category

Since the transition of the South Africa programme to an elimination agenda, the progress towards elimination across South Africa’s three malaria endemic provinces of KwaZulu-Natal (KZN), Limpopo, and Mpumalanga has varied (Fig. 1) [1]. The province closest to elimination is KwaZulu-Natal, which has seen a steady decline in local cases, from 102 in the 2010/2011 season (July 2010 to June 2011) to just 35 in the 2016/2017 malaria season (July 2016 to June 2017) [2]. With the possibility of achieving zero local transmission in KZN within reach, the KZN malaria control programme conducted a critical evaluation of its practices and protocols to identify potential challenges and priorities to achieving elimination [3, 4].

Three fundamental questions were addressed in the evaluation: (1) How close is KZN to malaria elimination; (2) Are all systems required to pursue subnational verification of malaria elimination in place (as outlined by the World Health Organization); and (3) What priority interventions must be implemented to reduce local cases to zero? The 2017 World Health Organization (WHO) Framework for Malaria Elimination was used as the guiding document for the evaluation process [5]. This document details the tools, activities, and strategies required to achieve interruption of transmission, and describes the process for obtaining WHO certification of malaria elimination.

As the WHO framework does not prescribe the operationalization and specific evaluation of its recommendations, the KZN malaria programme developed a checklist of twenty-eight key requirements for elimination and their related impact indicators. This malaria elimination checklist was tailored to the local context and aimed at assessing the programme’s performance towards malaria elimination and its readiness for sub-national verification. This manuscript presents the findings from this evaluation and key recommendations to accelerate towards malaria elimination in KZN.

## Methods

### Development of the checklist framework

The WHO Framework for Elimination outlines six key strategies and interventions for malaria elimination with specific activities for each of those strategies [5]. The WHO Strategy and the respective KZN Checklist components are shown in Tables 1 and 2. Within these six key strategies, twenty-eight separate requirements for elimination were delineated, which detail specific items for emphasis. For instance, within the strategy for enhanced case management, specific requirements include, amongst other requirements: all cases receiving the appropriate first-line treatment; ensuring that RDTs are available at all levels, and active targeted testing following detection of a local or imported case in a receptive area. For each of these twenty-eight delineated requirements for elimination, forty-nine specific indicators were identified and calculated across time and geographies (see Additional file 1).

**Table 1:** WHO framework key strategies and interventions and their related KZN malaria elimination checklist component

WHO framework key strategy or intervention	KZN malaria elimination checklist component
Local stratification by malaria intensity	Target interventions based on fine scale mapping and stratification, with strategies aligned with WHO global technical strategy pillars
Enhancing and optimizing case detection and case management and role of quality assurance and reference laboratories	Enhance and optimize case management—testing, treating and tracking
Enhancing and optimizing vector control	Achieve optimal coverage of vector control interventions wherever strata are both receptive and vulnerable to malaria transmission
Surveillance	Increase the sensitivity and specificity of the surveillance systems to detect, characterize and monitor all cases (individual and foci)
Accelerating activities towards elimination	Tailor response based upon classification and status of the program efforts to investigate and contain transmission
Management and planning	Ensure appropriate management and planning

The KZN checklist components are subsequently divided into twenty-eight requirements, and each of these requirements are, in turn, divided into forty-nine indicators. All levels are supported by myriad guideline and policy documents

**Table 2:** Framework of the elimination checklist

Research question	Method of analysing the question
(1) How close is KZN to malaria elimination?	Analysis of key impact indicators, such as total number of local indigenous and local introduced cases, overall incidence, positivity rate and the ratio between local and imported cases Disaggregation of impact indicators over time and geographical areas
(2) Are all systems required to pursue subnational verification of malaria elimination in place?	Analysis of twenty-eight separate requirements and forty-nine specific process or outcome indicators
(3) What priority interventions must be implemented to reduce local cases to zero?	Inclusion of programmatic indicators such as the availability of policy documents, guidelines, and reports Availability of all the items and indicators which are specifically required by the WHO for certification

### Evaluation of programme performance against the checklist

**Fig. 2:**
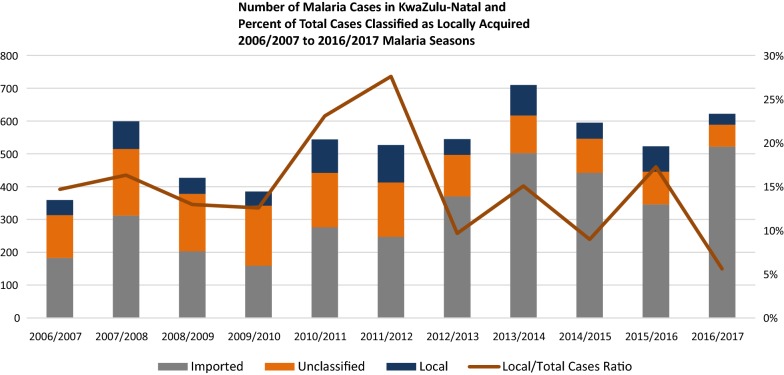
Cases per classification and percentage of total cases classified as locally acquired, between 2006/2007 and 2016/2017 seasons

To quantitatively and qualitatively evaluate programme performance against the KZN Checklist, monthly case data were extracted from the provincial and national malaria information systems (MIS) as well as the rapid malaria notification system, Malaria Connect (mobile phone data collection system). Additional data not captured in the surveillance systems (e.g. entomological data, population estimates were compiled from supplemental data sources used by the programme. The analysis was conducted at the municipal (sub-district) level, which is the lowest administrative unit available across all datasets in KZN (Fig. 2).

With consultation from the programme and with expert review from the National Department of Health and several technical experts who sit on the South Africa Malaria Elimination Committee, data from the previous 10 malaria seasons ending in the 2016/2017 season were used to calculate each indicator and semi-quantitatively rate performance into one of four categories:

- Fully implemented/well implemented.
- Partially done/somewhat implemented/activity needs to be strengthened.
- Not done at all/not implemented/poor performance.
- No data available to assess performance.

## Results

### Programme performance on collection of data for each indicator

Out of the 49 indicators in the checklist, 10 indicators (20%) were rated as fully implemented/well implemented. Eleven indicators (22%) were rated as partially done/somewhat implemented/activity needs to be strengthened, and 12 indicators (24%) were rated as not done at all/not implemented/poor performance. Sixteen indicators (33%) could not be calculated due to lack of data or missing data (see Additional file 2).

With regard to the supporting documentation and policies within the programmatic indicators, 18 (46%) were fully implemented/well implemented, indicating that sufficient guidance is available (e.g., within the National Strategic Plan or other standard operating procedures). A further 7 (18%) were rated as partially done, indicating somewhat insufficient or incomplete guidance, and 11 (28%) were not done at all, indicating no or highly limited guidance. For three items (8%), no data was available to assess performance.

### Programme performance on components and indicators towards elimination

#### Overall programme performance

**Fig. 3:**
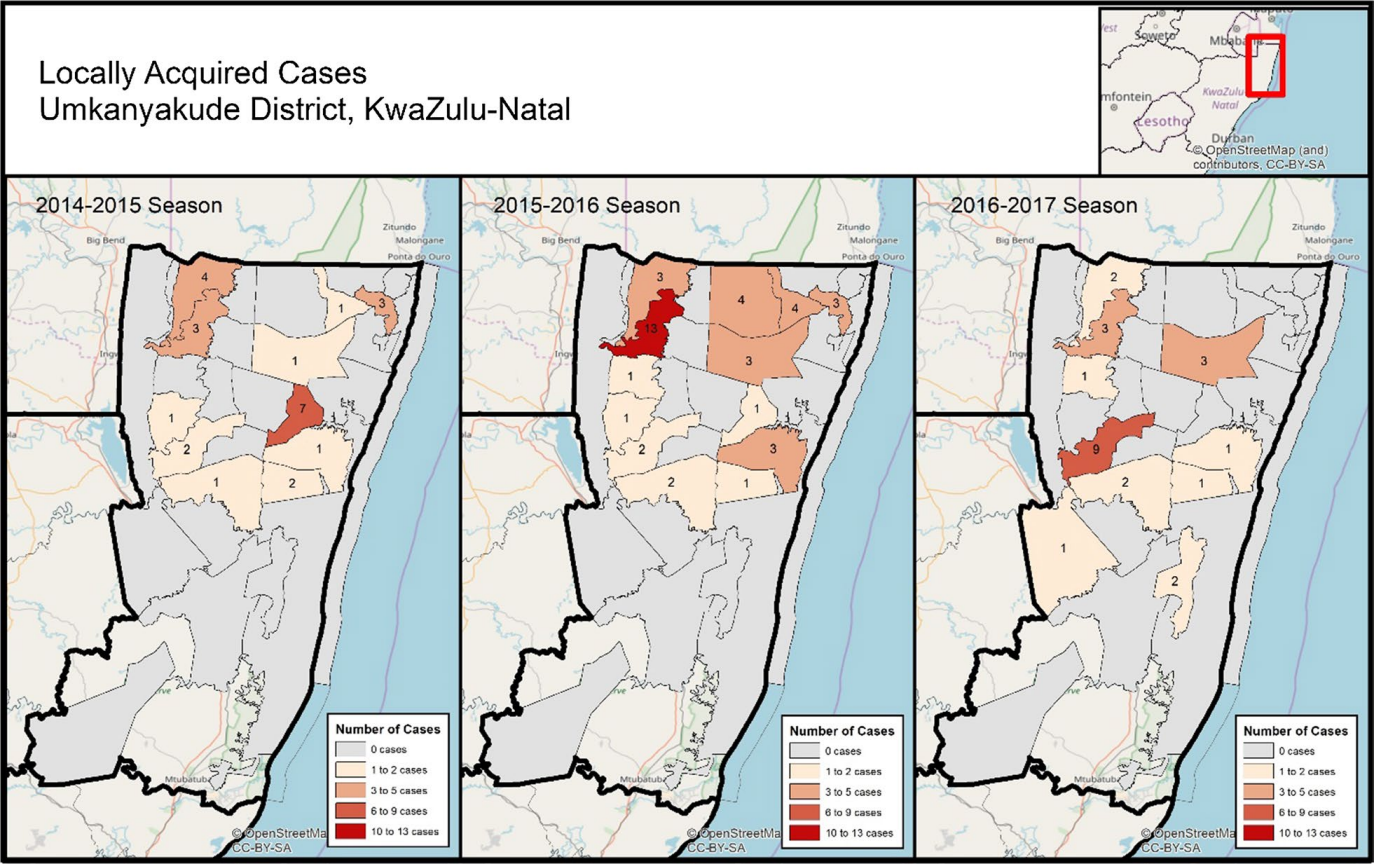
Map of cases per locality in KwaZulu-Natal for the 2014–2015, 2015–2016, and 2016–2017 malaria seasons

The KZN programme has seen a 68% reduction in the number of local cases since 2010/2011 season, with only 33 local cases reported during the 2016/2017 malaria season (Fig. 2). The reported local cases in 2016/2017 were highly clustered, occurring in only 4 of the 51 municipalities in KZN, (Jozini, Big Five False Bay, uPhongolo and Umhlabuyalingana). The municipalities are located in the Umkhanyakude and Zululand Districts in north-eastern KZN, close to the border with Mozambique. Unclassified cases (i.e., cases that could not be classified as local or imported) represent 11% (n = 53) of all confirmed cases in the 2016/2017 season, with 83% (n = 44) of these unclassified cases coming from the non-endemic District of eThekwini (Fig. 3) [2].

**Fig. 4:**
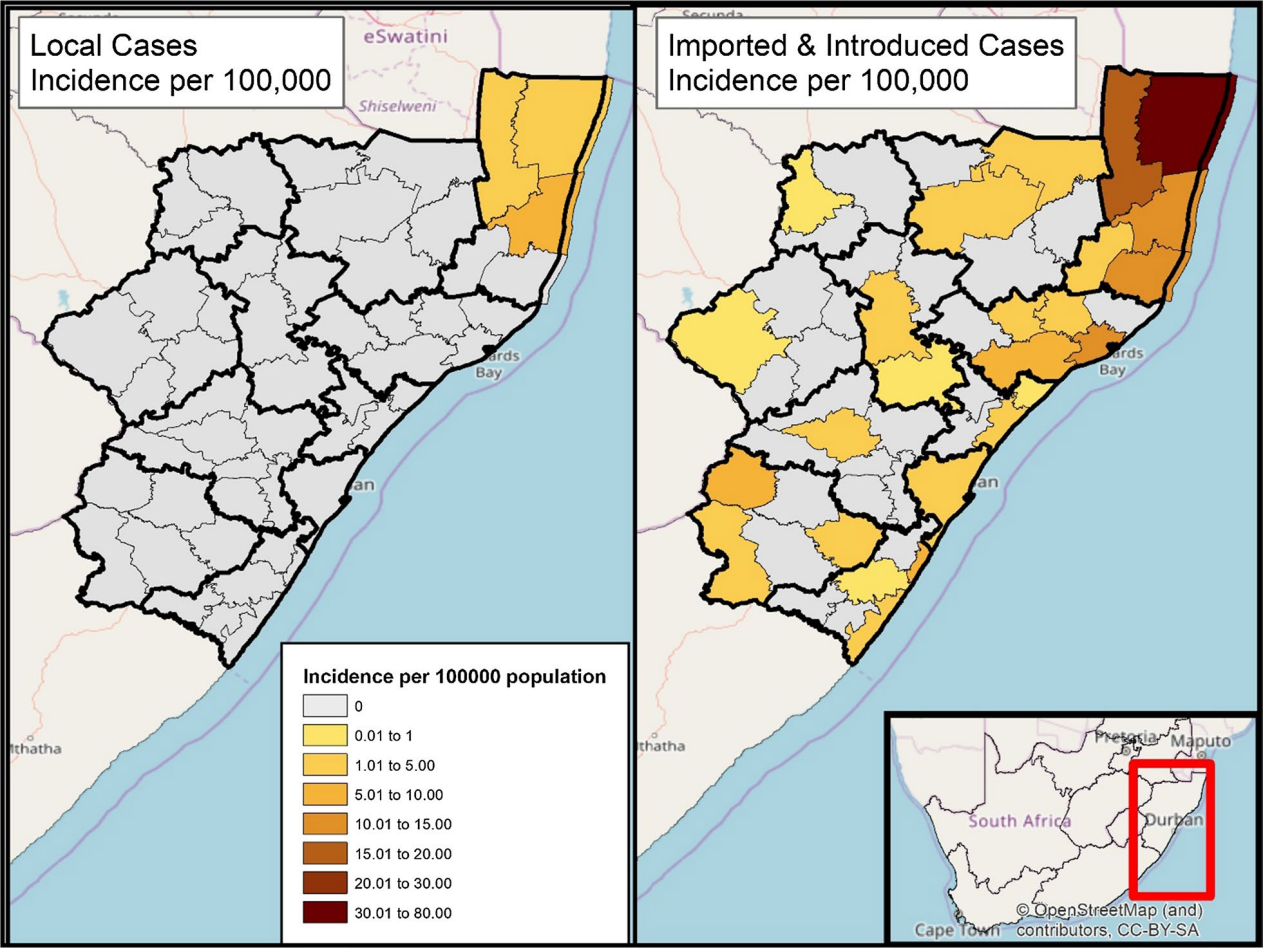
Map of Incidence per 100,000 population in KwaZulu-Natal, by classification as locally acquired or imported/introduced

The percentage of total cases classified as locally acquired has shown a downward trend, decreasing from 23% locally acquired in the 2010/2011 season to 6% in the 2016/2017 season. The change during this period can be attributed to both a decrease in local cases (102 to 35 cases) as well as an increase in imported cases (52 to 276 cases) (Fig. 4). In this same period, classification rates improved markedly, from 70% classified in 2010/2011 to 89% classified in 2016/2017. In 2016/2017 Umkhanyakude and Zululand were the only districts out of the 12 in KZN which reported local cases. Municipalities in Umkhanyakude reporting local cases included Hlabisa (0%), Jozini (24%), Big Five False Bay (50%), and Umhlabuyalingana (6%). Big Five False Bay was the only municipality that did not show a decline in the percentage of local cases over time.

#### Component 1: Target interventions based on fine scale mapping and stratification, with strategies aligned with WHO Global Technical Strategy pillars

A stratification exercise was conducted in 2012 by the national programme and was included in the 2012–2018 National Malaria Elimination Strategic Plan. Districts were classified into four strata based on local incidence: high (≥ 5/1000 pop. at risk), moderate (< 5 per 1000 pop. at risk), low (< 1/1000 pop. at risk) and very low (0 local cases). Within South Africa, stratification is mainly used to assess progress by reporting annually on the number of districts that moved from one stratum to another. It does not assign specific interventions based on malaria transmission intensity as recommended by the WHO 2017 Malaria Elimination Framework [5]. For this indicator to be rated as fully implemented/well implemented, specific packages of interventions will have to be recommended for each stratum, although this may not be directly applicable to KwaZulu-Natal due to all districts falling into the “very low transmission” category.

#### Component 2: Enhance and optimize case management—testing, treating and tracking

The provincial-level annual blood examination rate (ABER) is 1%, falling short of the WHO ABER target of 8% of the at-risk population tested. However, this masks successes at smaller scales. At the district level, ABER is very high in the endemic districts such as Umkhanyakude, which achieved an ABER of 13.5% in 2016 due to active case detection activities. In 2016, the programme expanded the case detection network to include both proactive and reactive case detection across the three endemic districts (King Cetshwayo, Umkhanyakude and Zululand) to improve ABER. Through these community-based case detection activities, 12 cases were discovered out of approximately 100,000 individuals tested proactively and 0 cases were discovered out of 2226 individuals tested reactively (Table 3).

**Table 3:** Testing of fevers during active case detection (reactive and proactive)

Districts	Total tested proactively by year				Total tested reactively by year			
	2014	2015	2016	2017	2014	2015	2016	2017
King Cetshwayo	12,231	11,439	10,135	15,099	68	119	22	0
Umkhanyakude	88,095	78,687	84,267	146,815	926	873	2113	3579
Zululand	7341	6260	5424	10,982	0	0	0	168
Total tested in all 3 districts	107,667	96,386	99,826	172,896	994	992	2135	3747
Total malaria positive in all 3 districts	15	6	12	7	0	0	0	0

Comprehensive data to assess per-facility testing rates to evaluate the passive case detection (PCD) system is limited as this data has only been collected since 2016. Between January 2016 and March 2017, only 65% of public and private health facilities currently offer on-site malaria testing and treatment services, and the per-facility ABER ranged from 0.00 to 1.47%, falling short of the WHO target. However, these data may show access to malaria care, as testing and treatment services for malaria are deliberately set at locations with risk of malaria. Of the 260 facilities reporting malaria cases in the current malaria season, 192 (74%) reported testing rates, but only 19 of those facilities (10%) reported testing rates consistently each month. Based upon the available data, at least 1749 suspected cases were tested at health facilities in the 2016/2017 season, with 41 malaria positive individuals detected, indicating a positivity rate of at least 2% among symptomatic individuals. However, this figure is most likely to be underestimated due to the underreporting of testing done at health facilities.

The Malaria Programme has its own microscopists who are not affiliated with the National Health Laboratory Service (NHLS) and rather sit under the MCP (Malaria Control Programme). Although some microscopists are part of the Proficiency Testing Scheme facilitated by the NHLS, QA/QC (Quality Assurance/Quality Control) is limited within the MCP. QA activities for the RDTs are not yet implemented. Shortage of key consumables and reagents, limited training of microscopists, and lack of participation of the malaria programme laboratories in a QA/QC programme were highlighted by two independent performance reviews [6, 7].

Conversely, the hospitals and large health facilities participate in the NHLS QA/QC programme. All NHLS laboratories performing malaria microscopy will participate in several quality assurance activities, including laboratory supervision, participation in EQA programmes, and rechecking of malaria microscopy slides, either within or between different laboratories. The parasitology unit at the National Institute for Communicable Diseases (NICD) manages the malaria QA/QC and PTS programmes for both microscopy and RDTs.

Treatment data were not captured electronically in the surveillance system prior to 2016, and only 70% of the cases reported in 2016/2017 had treatment data captured. For this analysis it was assumed that inpatient cases were those requiring treatment for severe malaria (artesunate or quinine). However, only 7% of inpatient cases received artesunate, and 38% received quinine. The low number of inpatients receiving artesunate may be explained as this treatment option was registered in South Africa in August 2017. Because of this recent registration, health facilities may have had challenges accessing the drug. Furthermore, “inpatients” may also refer to co-morbid patients with uncomplicated malaria cases which would require a different treatment.

#### Component 3: Achieve optimal coverage of vector control interventions wherever strata are both receptive and vulnerable to malaria transmission

Indoor residual spray (IRS) coverage (the number of structures sprayed out of total number targeted) has decreased from 84% in the 2010/2011 (n = 324,018) season to 53% in 2015/2016 (n = 323,090), and slightly increased to 66% in the 2016/2017 season (n = 487,130). It is worth noting that while the average IRS coverage at provincial level was very low in the 2016/2017 season, it ranged from 21% to 100% coverage in the localities targeted for IRS (n = 36), with 16 localities achieving coverage higher than 80%. Because GPS coordinates for locally-acquired cases were not routinely captured prior to 2016, it is unclear if the areas with cases were among the ones with low coverage in the next season; however, all targeted areas are based on transmission history as well as vector density.

Limited data are available to assess the entomological status in KZN. The identification of anopheline vectors collected in KZN is restricted to morphology, and further data on analyses to distinguish between species (especially for *Anopheles arabiensis* and *Anopheles funestus*) and infection rate are unavailable within the programme. No data was available to the programme to monitor the quality of the spraying (cone bioassays), insecticide resistance or behaviour and characteristics of the vectors for the period analysed. However, these indicators will be evaluated for the 2017/2018 IRS spray season.

#### Component 4: Increase the sensitivity and specificity of the surveillance systems to detect, characterize and monitor all cases (individual and foci)

**Fig. 5:**
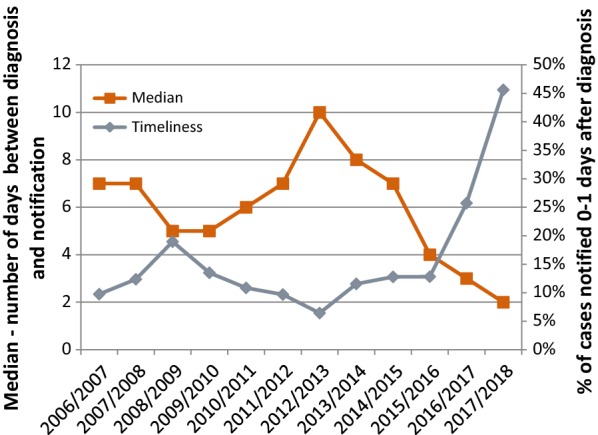
Timeliness of notification in KwaZulu-Natal from 2006/2007 to 2016/2017

The case confirmation rate and case reporting rate have been consistently above 99% since 2010/2011 season. Timeliness of notification has improved with the median number of days between diagnosis and notification decreasing from 10 days in 2010/2011 to 3 days in 2016/2017, when 54% of all cases were reported within 3 days from diagnosis and 91% of cases were reported within 7 days of diagnosis. However, in 2016/2017, only 21% of cases were reported on time according to national policies, which state cases must be reported within one day after the date of diagnosis (Fig. 5).

**Fig. 6:**
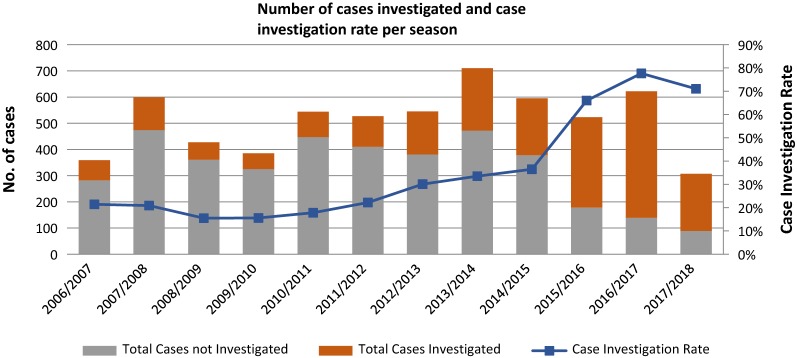
Case investigation (N and rate) in KwaZulu-Natal from 2006/2007 to 2016/2017

The case investigation rate has increased; 89% of reported cases were investigated in the 2016/2017 season as compared to 70% in 2010/2011 season (Fig. 6). Performance in the three remaining endemic districts is extremely strong, with completed investigations for 100% of reported cases in the 2016/2017 season. In Jozini and Umhlabuyalingana, the two municipalities with the majority of local cases (55% and 30% respectively for 2016/2017 season), investigations have been completed for 100% of reported cases for the past three seasons (Fig. 6).

**Fig. 7:**
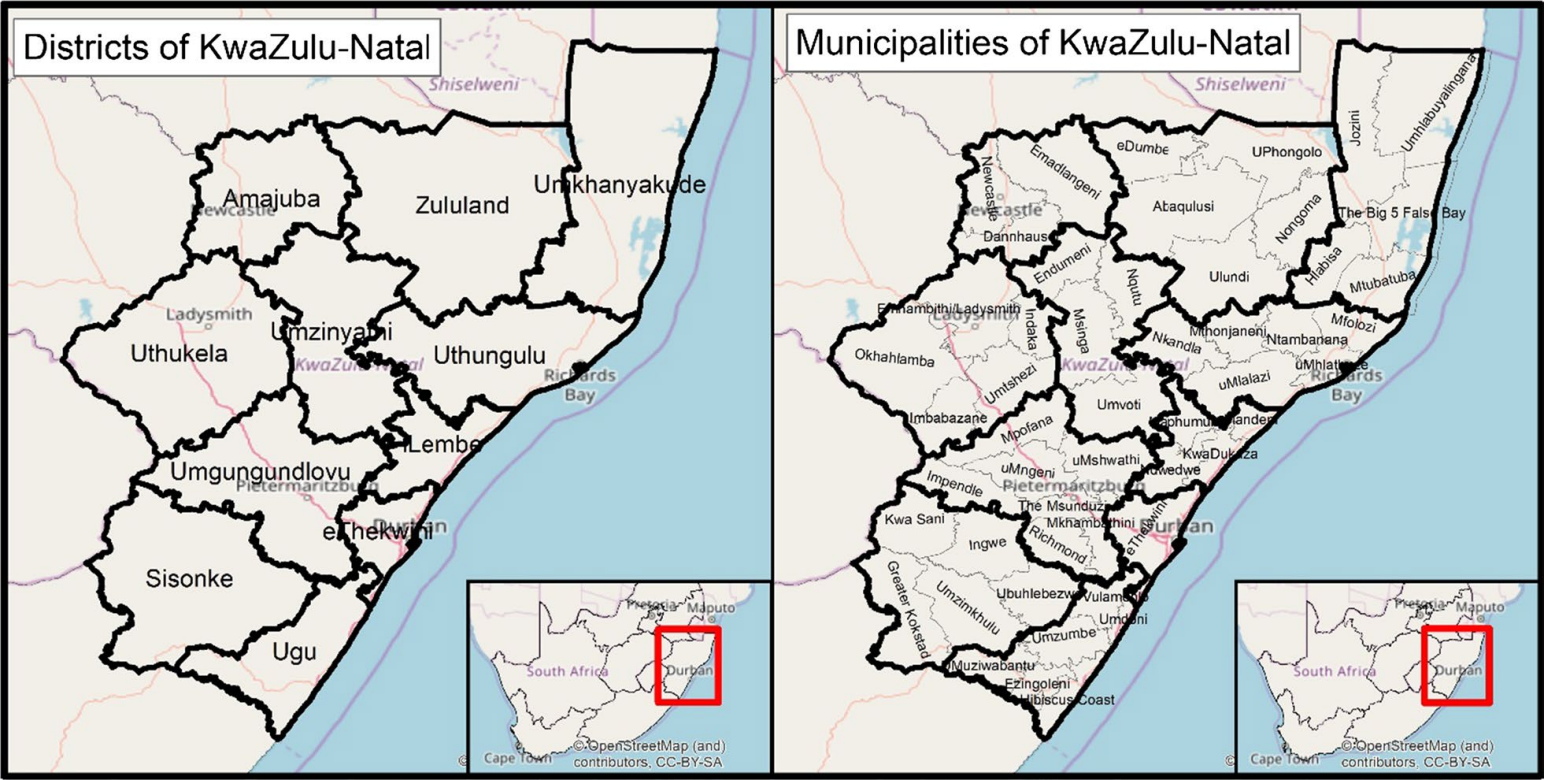
Districts and municipalities of KwaZulu-Natal

Out of all cases investigated in 2016/2017 season, only 3% had GPS coordinates which were able to be mapped due to various reasons ranging from GPS device accuracy and use as well as GPS data requirement policy. The quality of case geolocation data is also limited: out of the 50 geo-located cases available for the period from 2015 to 2017, eight points lie in Mozambique and two lie in Swaziland, casting doubt on the accuracy of the remaining 40 points. The programme is in the process of compiling a database of all household GPS coordinates in endemic localities which will enable any future cases to be more reliably geo-located (Fig. 7).

#### Component 5: Tailor response based upon classification and status of the programme efforts to investigate and contain transmission

All localities in endemic areas have been classified by endemic status based upon prior case data. According to this classification for the 2015/2016 season, there were 25 active foci (local cases in last year), 37 residual non-active foci (local cases between 1 and 3 years ago), and 370 cleared foci of transmission (no local cases in last 3 years). Localities were re-classified for the 2016/2017 season as 16 active, 39 residual non-active, and 377 cleared foci of transmission.

In the 2016/2017 season, nearly a third of local cases came from one active focus: Mamfene in the uMkhanyakude district. When this evaluation was conducted, no additional information is available on the potential drivers of transmission in the foci; however, a community survey comprised of diagnostic testing, a sociodemographic and travel history questionnaire, and entomological surveillance will be conducted by the programme in early 2018 to understand factors that may contribute to ongoing residual transmission.

#### Component 6: Ensure appropriate management and planning

Management at the KZN malaria programme encourages hiring, training and retention of staff. The programme is actively working toward building its entomological capacity by hiring an assistant entomologist and an entomology technician to increase the capacity of the department. While there are still many vacant posts (15% vacancy rate), the Monitoring and Evaluation officer position has now been filled after remaining vacant for many months.

The KZN programme is following the overall guidance from the 2012–2018 National Strategic Plan, which highlights the interventions and strategies that should be implemented in KZN. They also create an annual operational plan to ensure appropriate implementation of the activities, and host an annual review and planning meeting each year with all key malaria staff and stakeholders. The KZN programme also monitors critical metrics to validate data and adjust programme response. For example, all case investigation forms reported are crossed checked by an Environmental Health Practitioner (EHP) before being captured in the surveillance system. Foci investigation forms from the foci clearing programme are also reviewed and feedback is provided on a weekly basis. A Malaria Elimination Committee is currently in place and provides a semi-annual review of the progress and gaps.

## Discussion

The KZN programme has reached a historically low level of local malaria transmission, and is approaching elimination. By robustly analysing the provincial surveillance data and ranking the performance of the programme using the malaria elimination checklist, the programme was able to objectively examine shortcomings and challenges with regard to the planning, implementation and monitoring of their interventions. Furthermore, the KZN Checklist has enabled examination of the programme holistically, and highlights the progress that has been made to date in an unbiased and data-driven manner.

One of the most critical information gaps identified through the Checklist process was the classification and geolocalization of the cases by the programme. Contributing factors to this gap are the ability to differentiate between indigenous local and introduced local cases, the operational capacity of the malaria programme to classify cases in non-endemic districts, and the quality of case investigation data. With only 33 local cases in the 2016/2017 season and the very high importation of malaria cases from other provinces and neighbouring countries, it is assumed that a high percentage of the local cases identified by the programme are introduced. However, this differentiation is a new recommendation from WHO [5], and has not yet been operationalized by the programme. It is urgent that the programme get guidance on how to differentiate indigenous from introduced cases in order to assess how close the programme is to elimination.

Although it is extremely hard for the malaria programme to extend its reach to non-endemic areas, the WHO framework requires that all cases be notified, investigated, and classified by their most likely source of origin. Thus, while it is likely that those cases reported from non-endemic areas are imported, the malaria programme does not have proof of the source of infection and therefore is required to classify the case as locally acquired since these cases are reported through separate channels and do not have completed case investigations. Collecting travel history and other information directly at the time of diagnosis would help the programme reduce the number of ‘unclassified’ cases from non-endemic districts and is underway with the introduction of a new notification form across the province, in both endemic and non-endemic areas. The programme is also looking to partner with environmental health practitioners from non-endemic districts to support case investigation activities. Along with missing travel history data, the review of the case investigation forms showed that data completeness can be improved, particularly for GPS coordinates of cases. As KZN approaches elimination, it is critical that programme staff not just count local cases, but rather take proactive efforts to discern causes of ongoing transmission, and to appropriately respond when necessary [8, 9].

Another gap elucidated through the checklist is the timeliness and completeness of reporting. According to the South Africa’s National Health Act 61 of 2003, malaria is a notifiable disease, and thus all malaria cases should be reported within 24 h of diagnosis [10]. All public health facilities in endemic districts should participate in the surveillance system and report all malaria cases through standardized pathways, namely the case notification form and the Malaria Connect system, which allows health care workers to immediately report a case via any mobile phone free of charge. In non-endemic districts, health facilities should report through the national form for notifiable medical conditions. While the timeliness of notification of the cases has improved over time, there is still room for improvement. With the phased roll out of Malaria Connect beginning in 2015, timeliness should continue to improve [2, 3]. Reporting of private health facilities into the surveillance system also needs to be improved.

It is important to note that data available to evaluate passive testing rates is limited. The programme uses tally sheets of RDT consumption from health facilities to track this indicator; however, these tally sheets are not consistently completed and are not captured in the surveillance system. Based on the few data points currently available, it appears that testing at the health facilities is very low; however, this cannot be accurately determined due to the lack of available testing data. The limited testing during passive case detection is compensated in endemic areas by expansive active and proactive case detection; nonetheless effort must be made to better understand passive case detection in order to fully monitor case management practice and ensure prompt treatment of all infections. KZN has a very strong active case detection programme, with a high number of people tested, high ABER, and low positivity rates and incidence in the municipalities that have reported local malaria cases in the last few seasons. Endemic localities are achieving the WHO recommended targets for ABER figures of 8% of the at-risk population tested in active, residual active, new active and residual non-active foci; and 1–3% of the at-risk population tested in cleared up or new potential foci [3, 5].

Quality of diagnosis presents another critical issue, especially in the malaria programme laboratories that process blood smears collected during active case detection. Although laboratories in the main hospitals and health centres are participating in a strong QA/QC programme led by a reference laboratory (National Health Laboratory Service, NHLS) and NICD, the KZN Malaria Programme and its affiliated laboratories are not participating in an external QA/QC programme due to resource constraints, although this option is available. Moving forward, it is important to assess the value added by microscopy during active case detection and the potential of using new and more sensitive point-of-care diagnostics tools during active case detection (ACD), such as standard or Highly Sensitive RDTs. It would also be beneficial to look into opportunities to integrate the malaria programme laboratories within the larger laboratory system to ensure sustainability, and include the cost of participating in NICD quality control programmes into the programmatic budget [4, 5, 8].

With regard to quality of case management, the management of inpatient cases, assumed to be severe malaria cases, showed some limitation as only few patients received artesunate. This might be explained by the fact that, in the 2016/2017 season, only 7 out of 34 hospitals (21%) in KZN had artesunate in stock. This may also be partially due to the delays in registering artesunate in South Africa. It was also noted that some cases did receive the second-line treatment recommended by the National Department of Health (quinine) while the registration of artesunate was in process. With the registration completed in 2017, this situation will be monitored closely as it is likely to improve in subsequent malaria seasons.

Vector control is another an area for improvement, as the IRS spray coverage has been low with 66% of target covered in the 2016/2017 season and only 53% of target covered in the 2015/2016 season. However, the programme has performed well by being able to maintain a high number of structures targeted for spraying despite decreasing local cases year on year. Additionally, coverage in priority areas has shown to be high (> 80%). A shortage of insecticide may have contributed to the low coverage rate in the 2015/2016 season, as targeted areas had to be re-prioritized during operations. A common theme across multiple spray areas and seasons is that many targeted structures are not sprayed because they were temporarily locked. There might therefore be a need to improve the health promotion and communication activities currently implemented to sensitize communities to the benefits and process of the seasonal IRS campaign; as well as finding innovative ways to reach households which are available at hours outside of the norm.

Vector control operations also have room for improvement, as there is limited data available on the quality of IRS, and there are major knowledge gaps with regard to entomological data. The KZN programme implements IRS as a core intervention, but the effectiveness of the spray campaign is not evaluated by monitoring relevant changes in vector characteristics (susceptibility, density, and behaviours in adult population). Identification of the anophelines collected in KZN is restricted to morphological identification only. Further analyses to distinguish between species (necessary for *Anopheles arabiensis* and *Anopheles funestus*) and infection rate is unavailable. A review of published literature may shed some light on this and provide some species composition and resistance information; however, this may be restricted to the one focus where research takes place. This lack of entomological data may be partially explained by vacancies in the entomology team, with no entomology lead to support the data collection and analysis. Moving forward, it is important to have routine collection of entomological indicators [11].

The stratification and the response to identified foci are also affected by the lack of entomological data. The current foci classification is solely based upon transmission intensity and history because there is limited documented data of the receptivity of all areas. Thus, foci classification is not informed by entomological data or by intervention data. The WHO framework recommends that once a focus has been identified, an investigation is launched to delimit the area, characterize potential drivers of transmission, and characterize the populations at risk. The focus investigation should identify the main features of the location, including the location of actual or potential breeding sites, likely vectors and, if possible, insecticide susceptibility and behaviour [5, 11]. This will only be possible once the entomological team is fully operational.

The checklist also assessed whether the systems were in place for sub-national verification and certification. While the programme has many of the supporting policies and documents required for certification, key annual reports are outstanding and are not compiled annually. In addition, some critical gaps still exist including the lack of protocol to classify a case as ‘indigenous’ or ‘introduced’, poor insight into the activities of the passive surveillance systems (such as number of people tested for malaria and number of anti-malarial stock-outs occurring), the lack of a strong QA/QC programme for the laboratories, and overall gaps in case and entomological surveillance and reporting of data. WHO certification requires a strong surveillance system with at least 10 years of malaria surveillance reports, a national malaria case register with case investigation forms for at least the past 5 years and a foci register with full information about malaria foci for the past 5 years [5]. Therefore, it is important to build and fully operationalize these systems as soon as possible as they will not only facilitate achieving elimination, but will be necessary to obtain elimination certification from the WHO. This gap has been recognized by the National Department of Health, who are leading development of a comprehensive national MIS, however, ongoing commitment is required to ensure essential data is complete, fully reported, and utilized when actionable at the provincial level.

Finally, while the number of local cases has been decreasing since 2010/2011 season, the number of imported cases has been increasing. It is quite likely that Kwa-Zulu Natal will continue to report imported cases until elimination has been achieved and sustained in the other provinces of South Africa and neighbouring countries. The permanent risk posed by importation indicates that even after realizing malaria elimination in KZN, resources must remain available in order to prevent reintroduction in KZN. Cross-border collaborations such as the MOSASWA (Mozambique, South Africa and Swaziland) border surveillance units which test and treat along the shared borders need to be strengthened as the first line of defence against importation and reintroduction.

## Conclusion

Moving forward, the KZN malaria programme will be addressing the key issues identified through the Checklist. An action planning exercise took place in July 2017 to assign targeted activities to improve each indicator rated as poor or missing. A monitoring and evaluation framework with timelines and targets was assigned to each activity to monitor progress. In addition, multiple dissemination meetings were held with both the senior management in the province and field staff to ensure ownership of the checklist and its action plan at all levels. Monthly update reports on the progress of the monitoring and evaluation framework have been requested from the KZN Malaria Information Officer, and the entire checklist is to be updated on an annual basis.

From a national perspective, the checklist has been rolled out to both Mpumalanga and Limpopo provinces in South Africa to assess the gaps present and establish a holistic picture of the country’s malaria elimination progress and standing. It is important to note that, while the malaria programme is committed to eliminating malaria, it will require continuous support from the government and partners to ensure that human and financial resources are available not just to achieve elimination but to sustain elimination and prevent reintroduction. As South Africa reviews its malaria programme in 2018, the checklist will be used in all three endemic provinces to identify critical shortcomings. Prioritized recommendations will then form the inputs to the updated National Strategic Plan for malaria elimination post-2018.

## Additional files

**Additional file 1.** Checklist components and their descriptions.

**Additional file 2.** The KwaZulu–Natal checklist for malaria elimination and status of each requirement.
