# Supplementary material for: Assessing Kwa-Zulu-Natal’s progress towards malaria elimination and its readiness for sub-national verification
Source: Malar J. 2019 Apr 1;18:108. doi: 10.1186/s12936-019-2739-5 (PMC6444529; doi:10.1186/s12936-019-2739-5)
Supplement: Supplementary file 1 — Additional file 1. Checklist components and their descriptions. [file 12936_2019_2739_MOESM2_ESM.docx]

# Additional File 2. The KwaZulu-Natal Checklist for Malaria Elimination & Status of Each Requirement

*KZN Status within the checklist table is colour coded as follows:*

|  | Fully implemented / well implemented |  |  | Not done at all / not implemented / poor performance |
| --- | --- | --- | --- | --- |

|  | Partially done / somewhat implemented / activity needs to be strengthened |  |  | No data available to assess performance |
| --- | --- | --- | --- | --- |
|  |  |  |  |  |
|  |  |  |  |  |

| **Requirements for elimination** | **Indicators** | **Requirement for WHO Certification** | | **KZN Status** |
| --- | --- | --- | --- | --- |
| **Overarching Goal: Achieve and maintain interruption of local mosquito-borne transmission (reduction to zero incidence of indigenous cases) of malaria parasites in Kwa-Zulu Natal**  *(Cross-component, overall indicators)* | | | | |
| Requirements for elimination | Indicators | | WHO Required? | Status |
| Achieve and maintain interruption of local mosquito-borne transmission (reduction to zero incidence of indigenous cases) | Number of indigenous local cases | | X |  |
|  | Number of introduced local cases | |  |  |
|  | Number of local cases | | X |  |
|  | Test Positivity Rate | |  |  |
|  | Incidence (local introduced/local indigenous / imported) | |  |  |
|  | Prevalence | |  |  |
|  | Ratio local / imported | |  |  |
|  | Rc/R0 | |  |  |
| **COMPONENT 1: Target interventions based on fine scale mapping and stratification, with strategies aligned with GTS pillars** | | | | |
| Requirements for elimination | Indicators | | WHO Required? | Status |
| Stratification at the lowest geographical level for operational decisions | Geographic units stratified and classified according to transmission intensity | |  |  |
|  | Geographic units stratified and classified to identify receptive areas where malaria transmission has already been curtailed by the use of interventions | |  |  |
|  | Appropriate package of interventions determined to apply to different areas based on the result of the stratification | |  |  |
|  | Stratification and package of interventions regularly reassessed | |  |  |
| **COMPONENT 2: Enhance and optimize case management - testing, treating and tracking** | | | | |
| Requirements for elimination | Indicators | | WHO Required? | Status |
| Passive case detection covers the whole population, and all public health institutions diagnose and treat malaria | Proportion of the HFs providing malaria case management services:  Number of health facilities that are testing and treating malaria Number of health facilities | | X |  |
| Reach of the passive case detection network extended / Regular house-to-house visits in areas with inadequate PCD | ABER, APR, and API from Active Case Detection | |  |  |
| Continued screening of mobile or migrant populations with high risk of importing malaria | Population at risk of importation screened Population at risk of importation | |  |  |
| Active targeted testing following detection of a local or imported case in a receptive area | Active Case Detection screening: Number of cases tested (screened)  Number of cases investigated  Active Case Detection positivity: Number of cases detected (confirmed)  Number of cases tested | |  |  |
| Cases receive full treatment | Treatment rate =  Number of cases receiving effective treatment Number of cases confirmed | |  |  |
| Cases followed up at recommended intervals | Treatment follow up= Number of cases receiving effective treatment followed up at least once Number of cases receiving effective treatment | |  |  |
| RDTs available at all levels | RDT stock outs:  Number of health facilities that experienced RDT stock outs for more than 1 week in the past 3 months  Number of health facilities | | x |  |
| Treatment available through all available channels | Treatment stock outs:  Number of health facilities that experienced ACT stock outs for more than 1 week in the past 3 months  Number of health facilities | | X |  |
| Sufficient testing is occurring | ABER: Number of cases tested Population at-risk | | X |  |
| RDT and microscopy are quality assured | Score on the QA checklist | | X |  |
| Supporting structure/documents/policies | Definition of the roles of each service delivery channel | | X |  |
|  | National Reference Laboratory | | X |  |
|  | Reports of QA activities for diagnosis | | X |  |
|  | SOP for malaria diagnostics | | X |  |
|  | National antimalarial treatment policy | | X |  |
|  | Drug efficacy monitoring integrated into the National case surveillance system | |  |  |
| **COMPONENT 3: Achieve optimal coverage of vector control interventions wherever strata are both receptive and vulnerable to malaria transmission** | | | | |
| Requirements for elimination | Indicators | | WHO Required? | Status |
| Achieve optimal coverage of IRS in areas that are both receptive and vulnerable to malaria transmission | Target Rate:  Number of structures targeted by IRS within an operational unit /  Number of structures within the operational unit | |  |  |
|  | Coverage rate:  Number of structures covered by IRS within an operational unit  Number of structures targeted by interventions within an operational unit | |  |  |
| IRS established and maintained as a rapid response to clear foci | Percentage of active and residual non-active foci protected by IRS: Number of active and residual non-active foci protected by IRS Number of active and residual non-active foci | |  |  |
| Quality of IRS is monitored | Cone bioassays, IQK, QA, supervisory visits | |  |  |
| Interventions evaluated by monitoring relevant changes in vector characteristics (susceptibility, density, and behaviours in adult population) | Susceptibility testing data (WHO and cone bioassays to determine resistance in local populations) | |  |  |
|  | Indicators on abundance of active larval habitats, change in adult/larval vector composition/density | |  |  |
|  | Adult vector behaviour data (resting and feeding) | |  |  |
|  | Sporozoite infection rate, parity rate from wild caught females; from this the inoculation rate, longevity and vectorial capacity can be derived | |  |  |
|  | Entomology data available in the electronic surveillance system and used for decision making | |  |  |
| Supporting structure/documents/policies | Geo-positioning of houses sprayed for the evaluation of intervention coverage and effect | |  |  |
|  | Geo-positioning of vector breeding sites for the evaluation of intervention coverage and effect | |  |  |
|  | Implementation of IVM that is evidence based, and uses WHO recommended strategies | |  |  |
|  | Annual report of entomological and vector control activities | | X |  |
| **COMPONENT 4: Increase the sensitivity and specificity of the surveillance systems to detect, characterize and monitor all cases (individual and foci)** | | | | |
| Requirements for elimination | Indicators | | WHO Required? | Status |
| All suspected cases are tested | Testing rate: Number of cases tested  Number of cases suspected | |  |  |
| All tests are recorded | Proportion of health facilities reporting on their RDT consumptions: Number of HF filling and reporting their RDT testing log Number of HF | | X |  |
| All confirmed cases are reported | Reporting rate:  Number of cases notified  Number of cases confirmed | |  |  |
|  | Confirmation rate: Number of cases confirmed  Number of cases reported | |  |  |
|  | Timeliness: Number of cases notified within time  Number of cases notified | |  |  |
|  | Completeness: Number of health facilities reporting  Number of health facilities expected to report | |  |  |
| Case investigation form is completed for each confirmed malaria cases (characterization of the case, demographic info, history of current illness, test/treatment, travel history) | Case investigation: Number of cases investigated Number of cases notified | |  |  |
|  | Form completeness: All important fields of the case investigation form appropriately filled in Case investigation forms | |  |  |
| All cases are classified | Case classification rate: Number of cases classified  Number of cases investigated | | X |  |
| Cases geo-located to improve knowledge of where transmission is occurring | Geolocation rate: Number of cases with geolocation  Number of cases investigated | | X |  |
| Supporting structure/documents/policies | Malaria is a notifiable disease by national legal requirements | | X |  |
|  | Guidelines and SOPs for malaria surveillance | | X |  |
|  | Staff at all levels trained to examine and evaluate surveillance data | | X |  |
|  | Case and focus investigation reports are quality controlled at the district and central level, with direct feedback to the person/team who prepared the report | |  |  |
|  | PCD - Surveillance components follow SOPs and compliance is monitored real time | |  |  |
|  | ACD - Surveillance components follow SOPs and compliance is monitored real time | |  |  |
|  | Classification of cases include the following options: imported, introduced, indigenous, relapsing/recrudescent, or induced | | X |  |
|  | Conservative classification in situation of uncertainty | | X |  |
|  | Computerized, geo-referenced database with all cases, including those outside the public health system, to support program management, analysis, and response. | | X |  |
|  | Repositories of all reports and records maintained at district and central levels | | X |  |
|  | National malaria case register with case investigation forms for at least the past 5 years | | X |  |
|  | All available malaria surveillance reports going back at least 10 years, three years of which show zero indigenous cases | | X |  |
|  | Recent published/unpublished study reports on malaria epidemiology and malaria vectors | | X |  |
|  | Documentation on health education  and community awareness | | X |  |
| **Component 5: Tailor response based upon classification and status of the program efforts to investigate and contain transmission** | | | |  |
| Requirements for elimination | Indicators | | WHO Required? | Status |
| All foci are investigated and classified | Proportion of investigated and classified transmission foci: Number of foci investigated and classified Number of foci | |  |  |
| All foci investigations are recorded | Proportion of foci investigations reported; Number of foci investigation reported  Number of foci investigated and classified | | X |  |
|  | Each foci investigation include: delineation, characterization of the area and populations at risk | |  |  |
| Teams prepare response plans based upon investigation results | Proportion of foci responded to with appropriate response intervention, as per national guideline | |  |  |
|  | A map is prepared to show the location of the households of cases, key geographical features, other habitations, health facilities, roads, and coverage of all interventions | |  |  |
| Supporting structure/documents/policies | SOPs determine timelines for initiation and completion of focus investigations | |  |  |
|  | Reclassification of foci is based on regular review (yearly or seasonally) | |  |  |
|  | Copies of case and focus investigation, situation and response plans are communicated to relevant local actors. | |  |  |
|  | A register of foci is maintained at district and national levels, and updated with new data and findings | | X |  |
|  | Full information about malaria foci in the 5 years leading up to the last indigenous case, with supporting maps (database on malaria foci investigations; foci register and analytical tables, maps) | | X |  |
| **Component 6: Ensure appropriate management and planning** | | | | |
| Requirements for elimination | Indicators | | WHO Required? | Status |
| Strong program management | Program structures encourage hiring, training, and retention of staff with core skill sets, and capable supply chain systems | |  |  |
| Constant monitoring of critical metrics to validate data and adjust program response | Availability of regular (at least once a year) review of the performance of the NMCP with regard to its NSP M&E framework | |  |  |
| Independent national malaria elimination advisory committee provides an external view of the progress and gaps | Availability of an independent national malaria elimination advisory committee | |  |  |
| Supporting structure/documents/policies | Situations assessment of epi, operational, and financial status of the program at the beginning of the planning period | |  |  |
|  | Strategic plan is available | | X |  |
|  | Elimination operational plan is available | | X |  |
|  | M&E plan is available | | X |  |
|  | Strategic plans are costed to facilitate resource mobilization | |  |  |
|  | Annual malaria program report | | X |  |
|  | Reports of independent committees on malaria | | X |  |
|  | Reports on inter-sectoral collaboration | | X |  |
|  | Reports of border coordination activities | | X |  |
|  | Enabling environment (political commitment, necessary legislation, and strategic partnerships) | |  |  |
