# Supplementary material for: Assessing Kwa-Zulu-Natal’s progress towards malaria elimination and its readiness for sub-national verification
Source: Malar J. 2019 Apr 1;18:108. doi: 10.1186/s12936-019-2739-5 (PMC6444529; doi:10.1186/s12936-019-2739-5)
Supplement: Supplementary file 2 — Additional file 2. The KwaZulu–Natal checklist for malaria elimination and status of each requirement. [file 12936_2019_2739_MOESM1_ESM.docx]

# Additional File 1. Checklist Components and their Descriptions

| Component |  | Description |
| --- | --- | --- |
| Component 1 | Target interventions based on fine scale mapping and stratification, with strategies aligned with WHO Global Technical Strategy pillars | Assesses whether operational units are stratified and classified according to transmission, whether each stratum receives an appropriate package of interventions, and if the stratification is regularly reassessed. |
| Component 2 | Enhance and optimize case management - testing, treating and tracking | Assesses the quality and the reach of passive and active case detection. The key indicators that are calculated include, amongst others, the proportion of health facilities providing testing and treatment services, annual blood examination rate (ABER), positivity rate, treatment rate and stock outs indicators. |
| Component 3 | Achieve optimal coverage of vector control interventions wherever strata are both receptive and vulnerable to malaria transmission | Assesses the implementation of the vector control interventions. Since the programme in KwaZulu-Natal only implements indoor residual spraying (IRS) the key indicators reviewed were IRS targets and coverage rates (coverage rate being the number of structures sprayed divided by the number of structures targeted), as well as entomological indicators on the quality of IRS and on vector characteristics. |
| Component 4 | Increase the sensitivity and specificity of the surveillance systems to detect, characterize and monitor all cases (individual and foci) | Assesses surveillance indicators including reporting rate, timeliness and completeness of reporting, and case investigation and classification rates. Indicators are calculated at the lowest operational level possible to assess the sensitivity and specificity of the surveillance system. |
| Component 5 | Tailor response based upon classification and status of the program efforts to investigate and contain transmission | Assesses whether the programme is investigating and classifying its foci, and using the surveillance data to inform response. Indicators reviewed included the proportion of investigated and classified transmission foci, reporting rate of foci investigation, and proportion of foci responded to with appropriate response interventions. |
| Component 6 | Ensure appropriate management and planning | Assesses the malaria programme’s governance and ability to hire, train and retain staff. |
